# 

**Published:** 1865-09

**Authors:** 


### CHICAGO MEDICAL JOURNAL.

Terms, $2.00 per Annum.

### CONTENTS OF THE SEPTEMBER NUMBER.

PAGE.

ORIGINAL COMMUNICATIONS.

Excision of the Os Calcis and Part of the Astragalus for Caries. Recovery with a Perfectly Sound and Useful Foot. By A. J. Baxter, M. D., of Chicago, Ill ........ 385

Clinical Lectures on Diseases of the Eye. By E. L. Holmes, M. D , of Chicago, Lecturer on Diseases of the Eye and Ear in Rush Medical College, &c.—Diseases of the Retina and Optic Nerve ........ 390

A Case of Compound Comminuted Fracture of the Humerus, and Simple Fracture of the Tibia and Fibula—Right Arm and Leg. By E. J Nugent, M. D., of Homer, Ill ........ 395

SELECTED.

Discussion in the Royal Medical and Chirurgical Society, on the Hypodermic Administration of Certain Medicines ........ 398

Arsenic Eating ........ 408

On the Vegetable Origin of Diamonds ........ 410

On the Dysmenorrhœa, Metrorrhagia, Ovaritis, and Sterility Associated with a Peculiar Form of the Cervix Uteri, and the Treatment by Division. By Robert Barnes, M. D ........ 411

On Diabetes and its Treatment. By Dr. H. Bence Jones, A. M., F.R.S. 416

EDITORIAL AND MISCELLANEOUS ........ 427

BOOK NOTICES AND REVIEWS ........ 42[illegible]

